# A Rare Cause of Acute Hepatopancreatitis in a Nepalese Teen

**DOI:** 10.1155/2018/8456503

**Published:** 2018-11-21

**Authors:** Swastika Adhikari, Ashish Lal Shrestha, Sanjay Raj Thapa, Amrit Ghimire

**Affiliations:** ^1^Department of Pediatrics, Grande International Hospital, Dhapasi, Kathmandu, Nepal; ^2^Department of Pediatric Surgery, Grande International Hospital, Dhapasi, Kathmandu, Nepal

## Abstract

**Background:**

Acute pancreatitis (AP) coexisting with acute hepatitis (AH) in children is uncommon. Moreover, a single bacterial cause explaining both the complications is even rarer. Despite familiarity with the usual presentation of enteric fever, atypical presentations can go unnoticed.

**Case Presentation:**

A 16-year-old previously healthy male presented to the emergency unit with recurrent swinging pyrexia, abdominal symptoms, and jaundice for a week. Blood work-up revealed deranged liver function tests (LFTs) and elevated pancreatic enzymes. Further assessment with imaging made a diagnosis of AH and AP without necrosis. Blood culture positivity for *Salmonella typhi* eventually confirmed the diagnosis.

**Conclusion:**

This is an uncommon presentation of an infection that is fairly common in our part of globe. Nevertheless, early suspicion and recognition is the key to timely management. Regular follow-ups are required to pick complications up early.

## 1. Introduction

Enteric fever is a common food-borne disease and a major global health concern. In most cases, the pathological agent is *S. typhi* (*Salmonella enterica* serovar typhi), a Gram-negative bacterium. However, the incidence of *S. paratyphi* A as an emerging cause is on the rise in endemic regions. An estimated excess of 26.9 million cases occur annually, with 1% mortality, the vast majority of which is witnessed in Asia [1]. Accurate estimates of disease in Nepal are difficult to obtain, limited by the diagnostic facilities that are scarce even within the city and virtually nonexistent in peripheries [2].

Salmonella infection can lead to diffuse involvement of reticuloendothelial system and uncommonly affect other organ systems like kidneys, heart, intestine, skin, muscle, nervous system, and pancreas [3, 4]. Rarer complications include rhabdomyolysis, hepatitis, renal insufficiency, pancreatitis, meningitis, myocarditis, pneumonitis, arthritis, osteomyelitis, and parotitis [5]. Finding a deranged LFT is not uncommon but enteric fever presenting primarily with AP and AH is certainly not an ordinary event [3, 6].

## 2. Case Report

A 16-year-old, previously healthy teen presented to emergency department with complaints of high-grade intermittent fever for a week followed by severe periumbilical pain with multiple episodes of loose stools, vomiting, and jaundice for 3 days. He did not have history of drug or alcohol abuse and never received blood transfusions in the past. He did not have history of recent travel or trauma.

On examination, he looked ill and toxic but was conscious and well oriented. He was icteric and mildly dehydrated although hemodynamically stable. Temperature was recorded to be 101°F. He was in mild respiratory distress with a respiratory rate of 32/min and SpO_2_ of 98% on O_2_ at 4 litres per minute via face mask. Abdominal examination revealed tenderness with guarding over the periumbilical region and hepatosplenomegaly. Rest of the systemic examination was unremarkable. The investigations revealed a deranged LFT with direct hyperbilirubinemia (total 4.6 mg/dl, direct 2.3 mg/dl) and elevated enzymes (ALP 273 *μ*/l, AST 214 *μ*/l, ALT 123 *μ*/l, and G-GT 294 *μ*/l). The pancreatic enzymes were also elevated (Amylase-548 *μ*/l and Lipase-415 *μ*/l), whereas renal function tests and hemogram were both normal.

**Figure 1:**
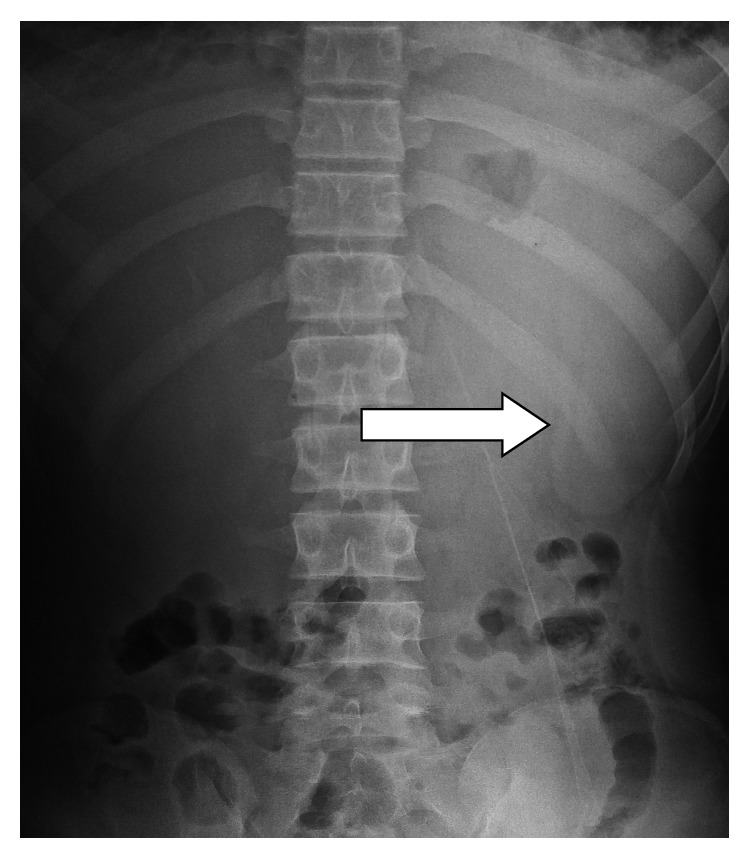
Plain abdominal radiograph with obvious outline of spleen associated with AP and AH.

**Figure 2:**
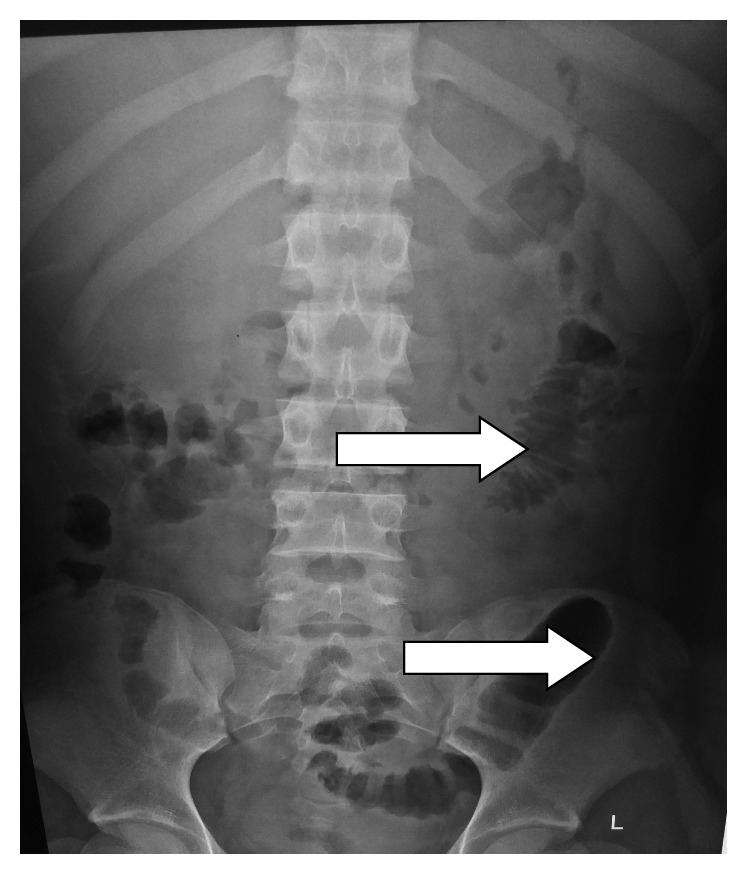
Sentinel loop sign and colon cut-off sign in a similar plain abdominal radiograph.

**Figure 3:**
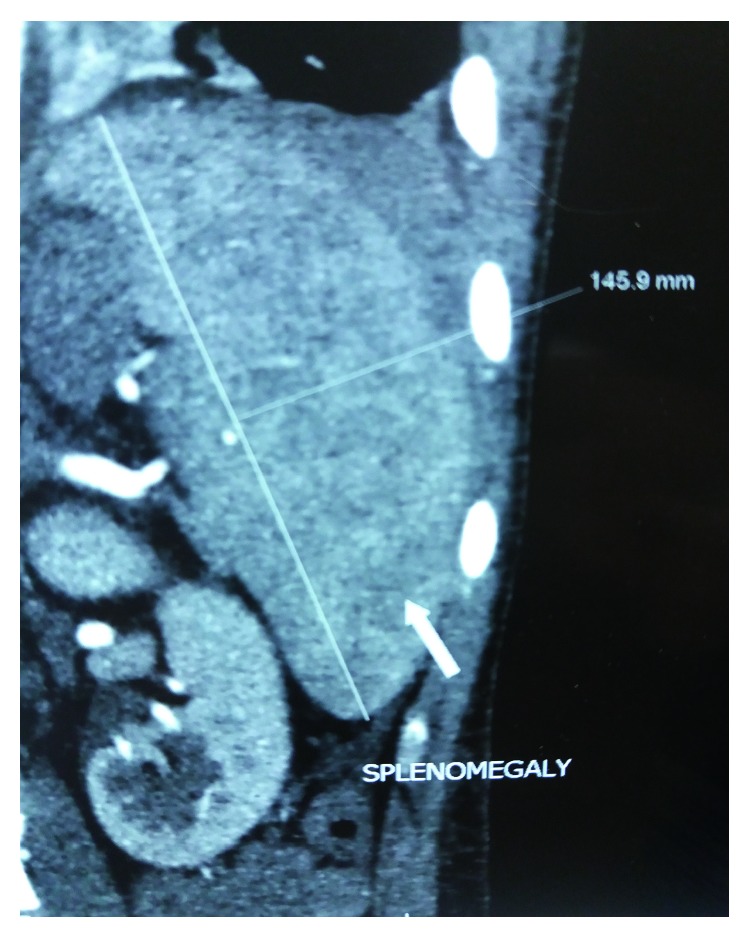
Splenomegaly 14.6 cm in CECT coronal view.

**Figure 4:**
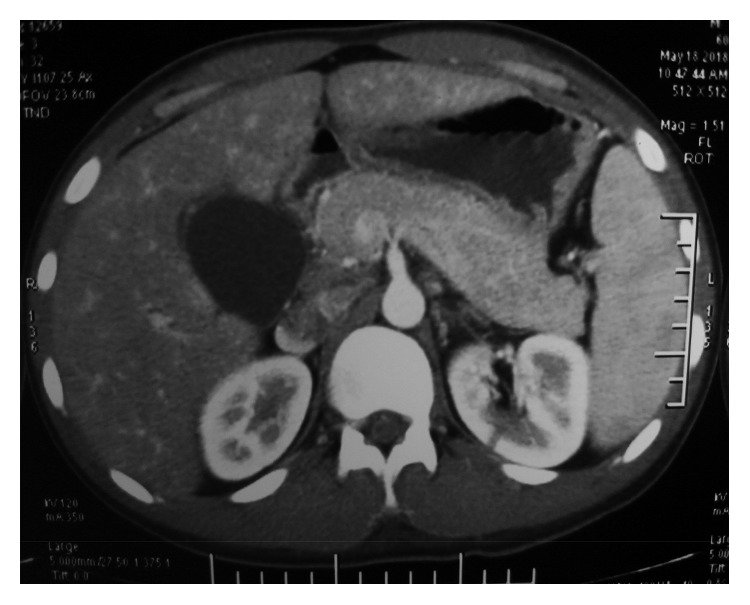
Bulky pancreatic tail and body with soft tissue stranding in peripancreatic fat.

**Figure 5:**
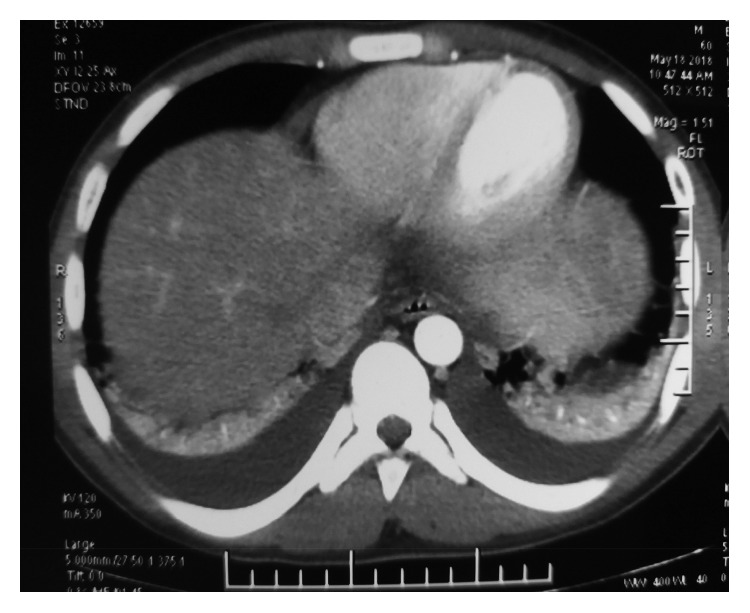
Bilateral basal atelectasis with mild pleural effusion.

**Figure 6:**
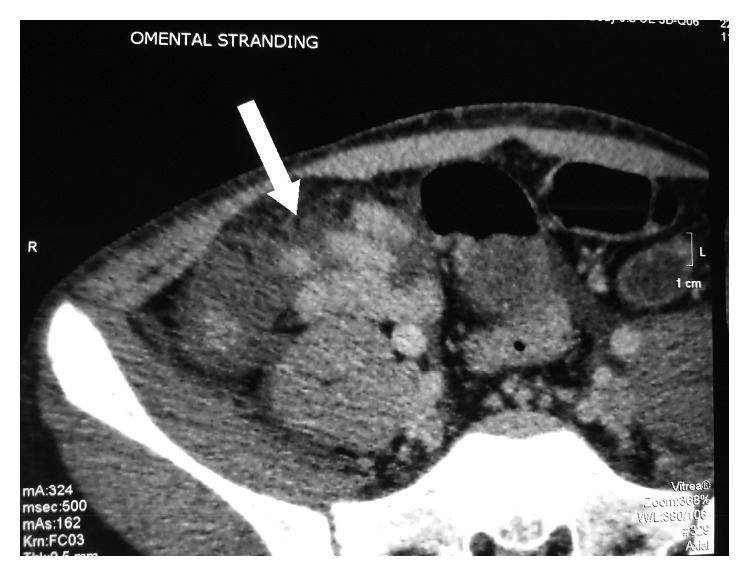
Omental fat stranding.

**Figure 7:**
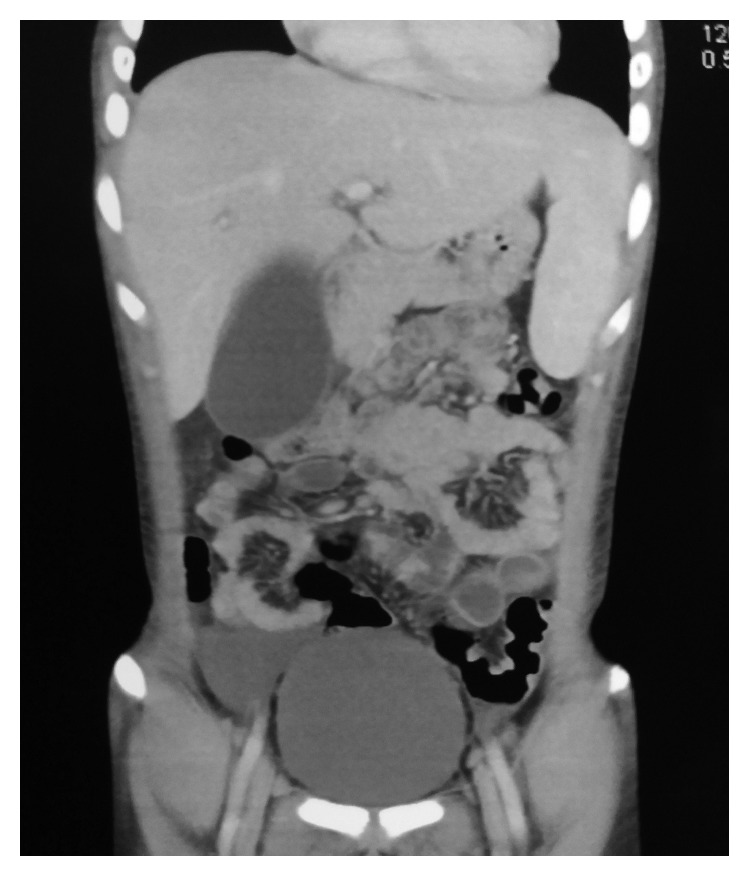
Similar findings as in Figure 4 in coronal view.

**Figure 8:**
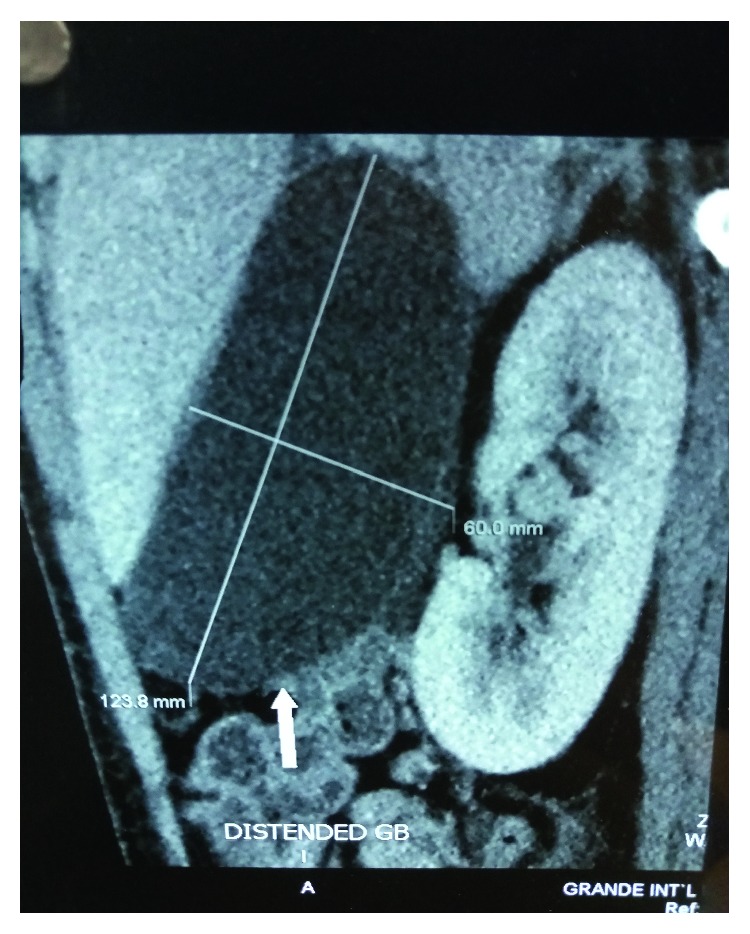
Distended gall bladder (12.3 × 6 cm) with minimal pericholecystic fluid.

**Figure 9:**
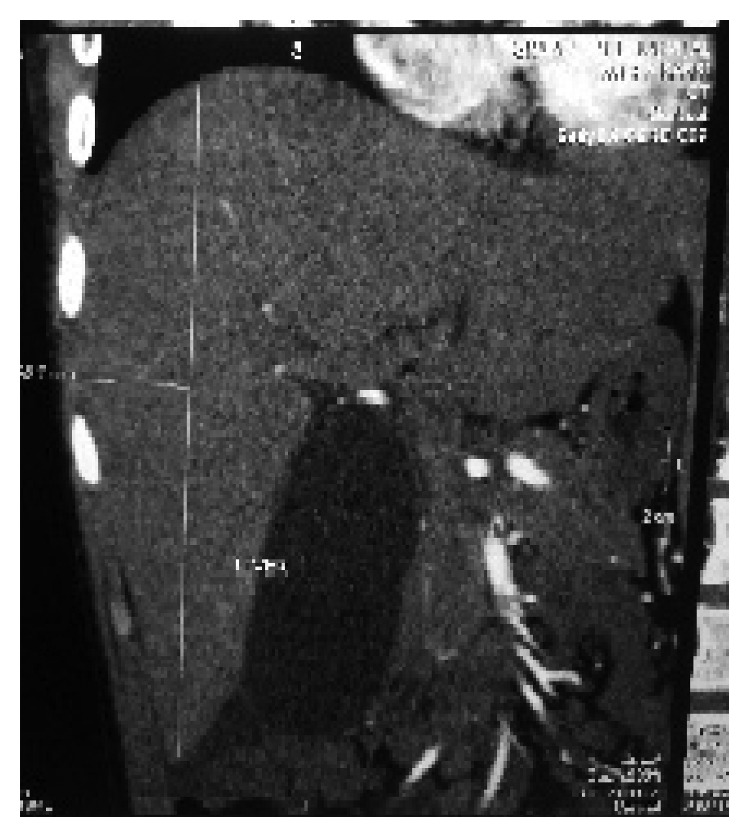
Hepatomegaly (20 cm along the longitudinal axis).

Plain abdominal radiographs showed colon cut-off and sentinel loop signs as shown in Figures 1 and 2. Ultrasound abdomen revealed hyperechoic pancreas, distended gall bladder, and splenomegaly. Further evaluation with an abdominal contrast enhanced computed tomography showed bulky pancreatic tail and body with mild soft tissue stranding in the peripancreatic fat, distended gall bladder with minimal pericholecystic fluid, and minimal ascites with hepatosplenomegaly. There was also bilateral minimal pleural effusion with subsegmental atelectasis of posterobasal segment of both lower lung lobes. Features favored acute interstitial pancreatitis: Modified Computed Tomography Severity Index (CTSI) score of 4 as shown in Figures 3–9.

Aided by Revised Atlanta Classification of AP 2012, a clinicoradiological diagnosis of interstitial edematous pancreatitis without pancreatic necrosis was made. Severity and mortality assessment done using Ranson's criteria at admission and within 48 hours predicted an associated mortality of 0–3%.

He was admitted to the Pediatric Intensive Care Unit (PICU), with a diagnosis of AP with impending septic shock and jaundice. Based on clinical findings, infective etiology was considered, and broad-spectrum antibiotics were initiated after sending blood culture and serological tests. He was kept nil by mouth, given intravenous fluid resuscitation, analgesics, and antipyretics.

After 4 days of intensive monitoring and supportive care, his fever and pain subsided. He could be gradually started on oral fluids and then on to fat-free diet. On day 6, he was stable to be transferred to the general ward, where he received a complete course of antibiotics for ten days and made an uneventful recovery.

**Figure 10:**
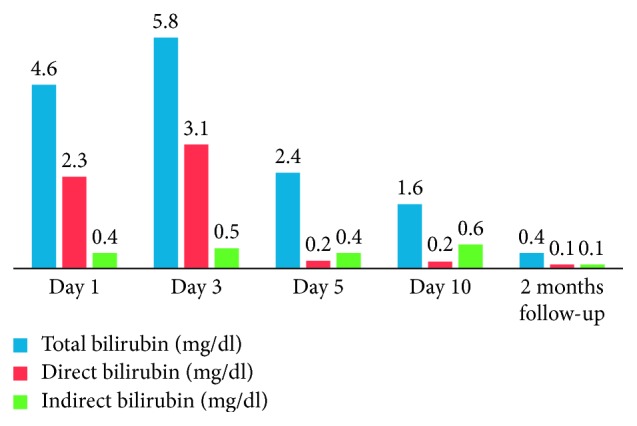
Profile of LFT with typhoid-induced AH. Profile of hyperbilirubinemia.

**Figure 11:**
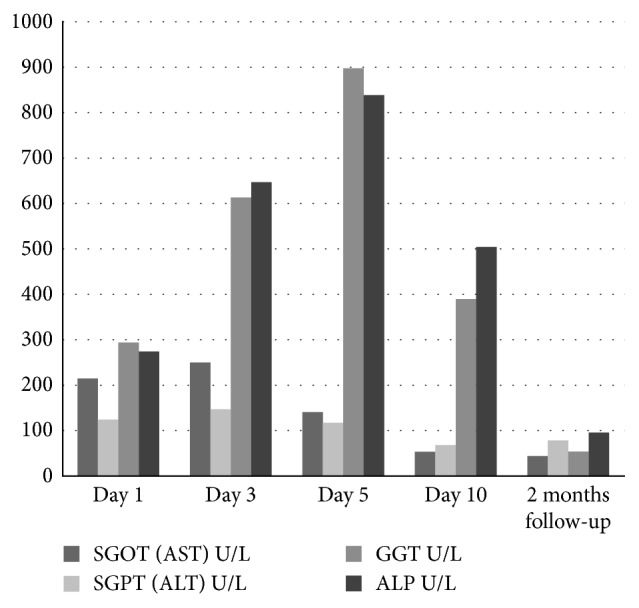
Profile of LFT with typhoid-induced AH. Profile of liver enzymes.

**Figure 12:**
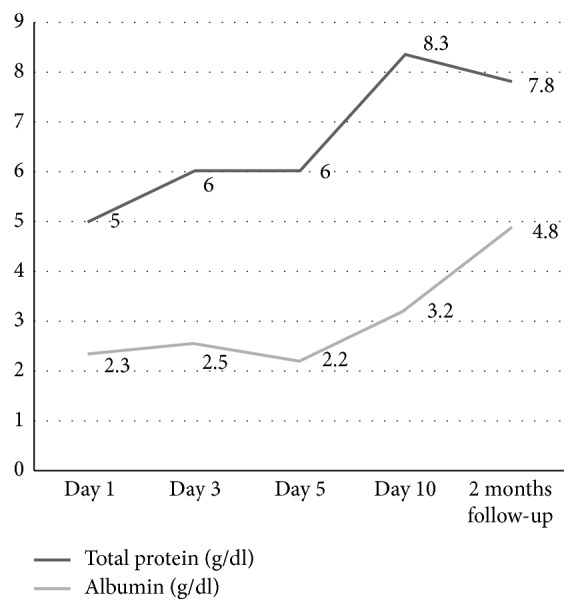
Profile of serum proteins with typhoid-induced AP.

**Figure 13:**
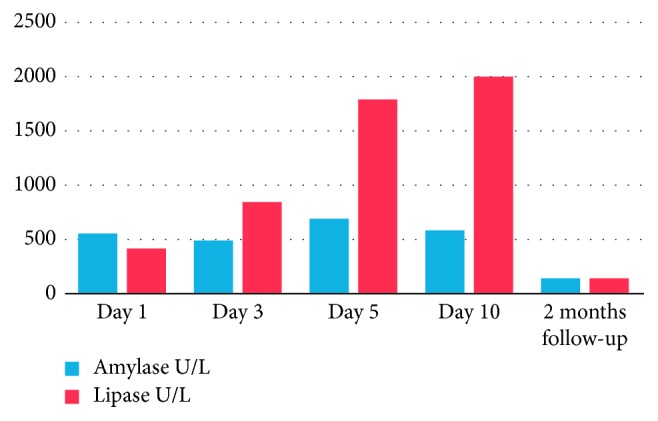
Profile of pancreatic enzymes with typhoid-induced AP.

LFT, serum proteins, and pancreatic enzymes were followed up till discharge and at 2 months, wherein gradual normalization in all was noted as shown in Figures 10–13.

Laboratory findings during hospitalization and at 2 months follow-up:

The serological tests for hepatitis B, C, leptospira, malaria antigen, and dengue were all reported negative, while the Widal test yielded positive for *S. typhi* “O” (1 : 320) and “H” (1 : 320). Based upon growth of “*Salmonella enterica serovar typhi*” on the blood culture 72 hours later, a final complete diagnosis of AP and AH secondary to *Salmonella typhi* infection was made.

## 3. Discussion

Childhood AP, although infrequent an event, is on the rise [7]. The diagnosis usually rests upon the findings of two of the following three: (1) characteristic abdominal pain, (2) elevated pancreatic enzymes (serum amylase or lipase >3 times the upper limit of normal), and (3) supporting evidence on cross-sectional imaging [8]. The etiological considerations in children vary from those in adults. For instance, in contrary to alcohol and gallstones that are commonly implicated in adults, in children, it is often the anatomic abnormality (choledochal cysts, pancreatobiliary maljunction, and pancreas divisum) followed by drugs, trauma, and infections [9]. Of infective etiologies, viral causes remain common while bacterial cause stands lower down in the list and precisely the reason why it can be missed [10].

Likewise, typhoid is not a common suspect to consider when managing a child presenting with AP and AH. But absence of usual suspects on imaging should arouse suspicion of rare causes for both.

The usual presentation of typhoid is that of a crampy pain around the umbilicus or over the right lower abdomen as opposed to the characteristic unrelenting pain of AP in the epigastrium radiating to the back and showing a postural relief in a typical knee elbow position. Similarly AH presents with pain over the right upper abdomen. The dilemma arises when all of these coexist.

Just as uncommon it is for typhoid to cause AP, equally uncommon it is to have typhoid causing AH [6]. To the best of our knowledge, this may probably be the first ever reported case of typhoid-induced AP and AH in the same child from Nepal. An extensive search in this regards in Pub Med, Medline, and Google yielded only 8 reports of typhoid-induced childhood acute pancreatitis in global literature amongst children below eighteen years of age, all of which have been summarized in Table 1.

Typhoid in most cases, in otherwise healthy children, presents with mild to severe diarrhea, nausea, and vomiting that generally subsides within 2–7 days. However, some may experience severe disease and present with sepsis and other complications. When it affects multiple organ systems, it is said to have a complicated course as was the case with our patient. Definitive diagnosis is based on culture with identification of *Salmonella* in stool or blood sample [1].

The mechanism of development of enteric pancreatitis with it is not well understood. The possibilities include (1) direct pancreatic localization of bacteria by hematogenous or lymphatic route, (2) transmural migration via the biliary tract and from the duodenum via the main pancreatic duct, and (3) toxin-induced response or immune mediated pancreatitis [16]. Likewise, the mechanisms leading to enteric hepatitis are again multifactorial [3]. The possible associated factors include virulence of the organism, delayed treatment, and poor health condition of the patients.

In our patient, the working diagnosis was based on clinicoradiological and biochemical grounds further substantiated by microbiology and eventual growth of “*Salmonella enterica* serovar typhi” on the blood culture 72 hours later. In view of impending sepsis, he was initiated on broad-spectrum antibiotics with aggressive supportive care and monitoring in the PICU that eventually resulted in a good outcome.

Regardless of its etiology, 30–75% cases of AP in children can be treated with this form of management with bowel rest and intravenous fluids with or without nasogastric decompression. Although, the available studies regarding antibiotic therapy are inconclusive, in those with AP secondary to Salmonella infection, aggressive use of antibiotics with supportive measures has yielded good results. Of these, in less than 5%, other complications may ensue that include recurrent pancreatitis, pancreatic pseudocysts (PPCs), pancreatic necrosis, and hemorrhagic pancreatitis [17].

The prevention of disease recurrence and complication seems to be the key issue in managing AP, as important is the regular follow-up [18].

Our patient had shown a remarkable improvement after 4^th^ PICU admission day with resolution of fever and abdominal pain. Following usual guidelines, we were able to start him gradually on oral feeds comprising fat-free diet. Approaching discharge, his hepatic and pancreatic enzymes were repeated and showed improvement in the former while the latter was still high. At 1 week and 2 months follow-up, he was clinically well. The enzymes repeated at 2 months showed pancreatic panel as amylase-133 and lipase-143 and LFT as bilirubin (total 0.4 mg/dl; direct 0.1 mg/dl) and liver enzymes (ALP 95 *μ*/l, AST 43 *μ*/l, ALT 77 *μ*/l, and G-GT 52 *μ*/l). He did not have symptoms to suggest or mandate further work-up for a PPC.

## 4. Conclusion

Acute hepatopancreatitis in children can have varied etiologies, different from those in adults. If the initial assessment is inconclusive, rarer causes need to be considered. Enteric fever as a possibility should be borne in mind in endemic regions.

## Figures and Tables

**Table 1:** Worldwide case reports of enteric fever-induced AP in children.

S. No.	References	Country	Year	Clinical data	Complications	Outcome
1	Herman et al. [4]	Morocco	1982	18 yrs/Female	AP	Recovered
			1984	16 yrs/Female		
2	Hearne et al. [11]	USA	1989	17 yrs/Male	AP	Details unavailable
3	Yacaman-Handal et al. [12]	Mexico	2000	4 yrs/Male	AP	Recovered
4	Asano et al. [13]	Japan	2007	4 yrs/Female	AP	Recovered
5	Martinez-Roig et al. [14]	Spain	2009	11 yrs/Male	AP	Recovered
6	Basak et al. [6]	Bangladesh	2015	17 yrs/Male	AP, AH, and pulmonary hypertension	Recovered
7	Snelling et al. [5]	Australia	2017	11 yrs/Female	Rhabdomyolysis and AP	Recovered
8	Roy et al. [15]	India	2017	5 yrs/Female	AP	Recovered

## Abbreviations

AP: Acute pancreatitis
AH: Acute hepatitis
LFT: Liver function tests
PICU: Pediatric Intensive Care Unit
PPC: Pancreatic pseudocyst.
